# The Ladder Rung Walking Task: A Scoring System and its Practical Application.

**DOI:** 10.3791/1204

**Published:** 2009-06-12

**Authors:** Gerlinde A. Metz, Ian Q. Whishaw

**Affiliations:** Canadian Centre for Behavioural Neuroscience, Department of Neuroscience, University of Lethbridge

## Abstract

Progress in the development of animal models for/stroke, spinal cord injury, and other neurodegenerative disease requires tests of high sensitivity to elaborate distinct aspects of motor function and to determine even subtle loss of movement capacity. To enhance efficacy and resolution of testing, tests should permit qualitative and quantitative measures of motor function and be sensitive to changes in performance during recovery periods. The present study describes a new task to assess skilled walking in the rat to measure both forelimb and hindlimb function at the same time. Animals are required to walk along a horizontal ladder on which the spacing of the rungs is variable and is periodically changed. Changes in rung spacing prevent animals from learning the absolute and relative location of the rungs and so minimize the ability of the animals to compensate for impairments through learning. In addition, changing the spacing between the rungs allows the test to be used repeatedly in long-term studies. Methods are described for both quantitative and qualitative description of both fore- and hindlimb performance, including limb placing, stepping, co-ordination. Furthermore, use of compensatory strategies is indicated by missteps or compensatory steps in response to another limb’s misplacement.

**Figure:**
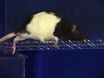


## Protocol

### Ladder rung walking test apparatus

The horizontal ladder rung walking test apparatus consisted of side walls made of clear Plexiglas and metal rungs (3 mm diameter), which could be inserted to create a floor with a minimum distance of 1 cm between rungs (see Fig. 1; Metz and Whishaw, 2003). The side walls were 1 m long and 19 cm high measured from the height of the rungs. The ladder was elevated 30 cm above the ground with a neutral start cage and a refuge (home cage) at the end. Because animals were habituated during training, the elevation of the apparatus was unlikely to cause anxiety. The width of the alley was adjusted to the size of the animal, so that it was about 1 cm wider than an animal to prevent the animal from turning around.

The difficulty of the task was modified by varying the position of the metal rungs. A regular pattern of the rungs allowed the animals to learn the pattern over several training sessions and to anticipate the position of the rungs (Fig. 1, Pattern A). An irregular pattern that was changed from trial to trial prevented the animal from learning the pattern (Fig. 1, Pattern B). For the regular arrangement, the rungs were spaced at 2 cm intervals. For the irregular pattern, the distance of the rungs varied systematically from 1 to 5 cm. Five templates of irregular rung patterns were used, so that the same patterns were applied to all animals to standardize the difficulty of the test and enhance comparability of the outcome (see results).

### Videorecording

A camera (Canovision, Canon Inc.) was positioned at a slight ventral angle, so positions of all four limbs could be recorded simultaneously. The shutter speed was set at 500 - 2000 s. The videorecordings were analyzed using frame-by-frame analysis at 30 f/sec.

### Behavioral training and test analysis

The animals were trained to cross the ladder from a neutral cage to reach their home cage, so the home cage with littermates provided the positive reinforcement for walking. All animals crossed the ladder in the same direction. No further reinforcement was given to motivate the animals to cross the ladder. All animals were trained and tested five times per session.

### Foot fault scoring

The qualitative evaluation of forelimb and hindlimb placement was performed using a foot fault scoring system as described earlier (Metz and Whishaw, 2003). Analysis was made by inspection of the video recordings frame-by-frame. Only consecutive steps of each limb were analyzed. Therefore, the last step before a gait interruption, such as a stop or a foot fault, and the first step after an interruption were not scored. The last stepping cycle performed at the end of the ladder was also excluded from scoring. Limb placement was scored in terms of limb placement on a rung and limb protrusion between rungs when a miss occurred.

The types of foot or paw placement on the rungs were rated using a 7-category scale (see Fig. 2). Foot or paw placement on the rung was rated according to their position and errors that occurred in placement accuracy.

**(0) Total miss.** 0 points were given when the limb completely missed a rung, i.e. did not touch it, and a fall occurred. A fall was defined as a limb deeply falling in-between rungs and body posture and balance were disturbed. **(1) Deep slip.** The limb was initially placed on a rung, then slipped off when weight-bearing and caused a fall. **(2) Slight slip.** The limb was placed on a rung, slipped off when weight bearing, but did not result in a fall nor interrupt the gait cycle. In this case, the animal was able to maintain balance and continue a coordinated gait. **(3) Replacement.** The limb was placed on a rung, but before it was weight bearing it was quickly lifted and placed on another rung. **(4) Correction.** The limb aimed for one rung, but was then placed on another rung without touching the first one. Alternatively, a score of 4 was recorded if a limb was placed on a rung and was quickly repositioned while remaining on the same rung. **(5) Partial placement.** The limb was placed on a rung with either wrist or digits of the forelimb or heel or toes of the hindlimb.  **(6) Correct placement.** The midportion of the palm of a limb was placed on the rung with full weight support.

When different errors occurred at the same time, the lowest of the scores was recorded. For instance, if a foot was first placed on a rung and then placed on another one in the same step (score 3), and then slipped and fell in-between rungs (score 1), a score of 1 was recorded. When a fall occurred, only the limb initiating the error was rated and none of the other limbs was scored until the animal had repositioned all limbs. Error scores of five trials were averaged for analysis.

### Foot placement accuracy analysis (number of errors)

The number of errors in each crossing was counted. Errors were determined based on the foot fault scoring system. An error was defined as each limb placement that received a score of 0, 1 or 2 points, i.e. an error represents any kind of foot slip or total miss. The number of errors and the number of steps was recorded for each limb separately. From these data, the mean number of errors per step was calculated and averaged for five trials. The second quantitative parameter analyzed was the average time needed to cross the entire length of the ladder task. Time measurement started after the animal was placed on the ladder and began walking the entire length of it. The time an animal spent in a stop was not included in the measurement.

### Forepaw digit score

The forepaw digit score was recorded when the paw was placed correctly with its midportion on a rung (i.e. score 6 in the foot fault scoring system). The degree to which the digits of the forepaw could be flexed around a rung was rated in a three-point scale (Fig. 2). The score was applied to the position with the maximum weight support, i.e. the forelimb was positioned vertically on a rung. The scores were given as follows: (0) Digits closed in an approximate 90-degree angle. (1) Digits closed in an approximate 45 degree angle. (2) Digits completely flexed around the rung. Scores of five steps were averaged and used for analysis.

### Figure legends


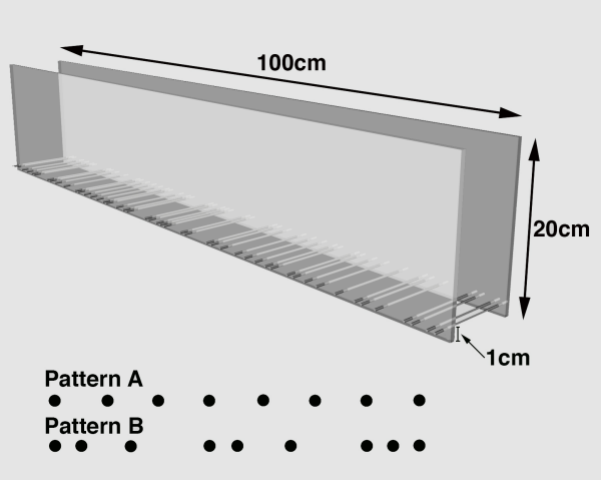
 ****

**Fig 1.** The skilled ladder rung walking test apparatus in the frontolateral view, with the specific measurements of the apparatus indicated. Two different rung patterns were used in the present study: Pattern A, regular rung arrangement, Pattern B, irregular rung arrangement. The irregular rung arrangement varied between consecutive trials.


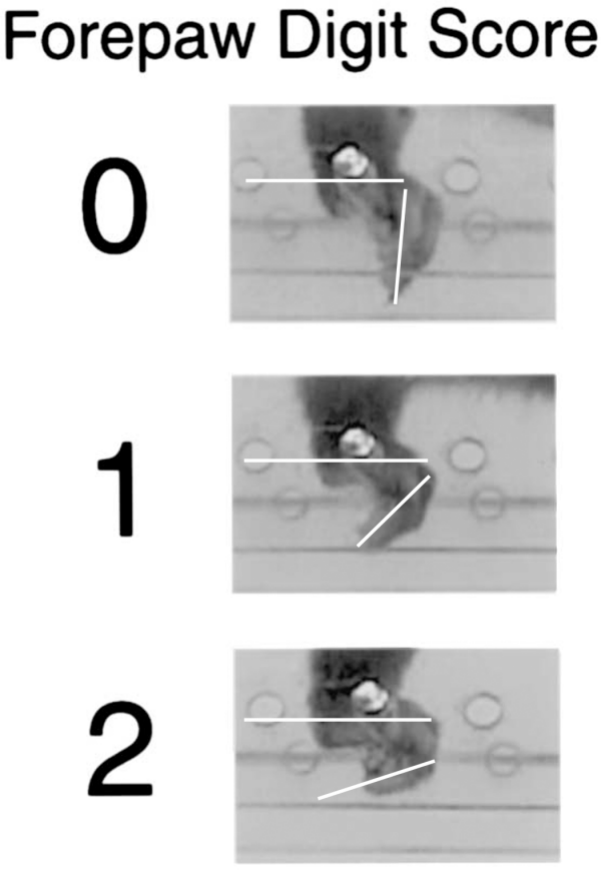


**Fig. 2.** Representative photographs illustrating the three categories of the digit score. Note the angle of the digits in relation to the forelimb when in full weight support.

## Disclosures

We have nothing to disclose.

## Discussion

The present study introduces the ladder rung walking task as a new test to assess skilled walking, limb placement and limb co-ordination. The ladder rung walking task allows discrimination between subtle disturbances of motor function by combining qualitative and quantitative analysis of skilled walking. The video analysis procedure can be used for a more descriptive account of impairments, and the simple rating scale described in this paper reveals any errors in limb placement and digit flexion when grasping a rung. Qualitative analyses counting the number of footfall errors made and the time needed to cross the apparatus. These measures allow an efficient and valid examination of foot placement accuracy and digit use.

There are a number of tests of walking, including tests of rotorod walking, beam walking, cylinder test, and gait analysis, and ground reaction force analysis (Muir, 2005). Each of these tests can usefully fragment limb placement for behavioral analysis. There are a number of strength for the present testing protocol, however. First, the test allows spontaneous walking to be examined, because the animals require no food or water deprivation for motivation. Second, stepping across the rungs requires precise foot placement and grasping, allowing for analysis of both stepping and grasping. Most importantly, the present test provides a challenge to ongoing locomotion because the spacing of the rungs is varied. The variation can be used to challenge ongoing walking and/or memory of the stepping patterns (McVea and Pearson,2007).

The strength of the ladder rung walking test is that it is sufficiently challenging to reveal subtle chronic impairments in both fore- and hindlimb use and unmask impairments that require forebrain control. In the irregular rung condition of the task, animals are not capable of anticipating the rung location and learning a specific gait pattern. Consequently, each step requires adjustment in paw placement, stride length, and in distribution of body weight. While normal animals are able to adjust to an irregular rung pattern within a few trials, this ability is limited in animals with motor system lesion. The irregular rung arrangement also requires that the animals adapt their regular limb co-ordination to changing rung distances. In order to adapt limb co-ordination, animals have to control their weight support to quickly correct for any limb placement error. After a unilateral lesion, animals partially compensate for placement mistakes by using the intact limbs for weight support. Consequently, the ladder rung walking test can measure compensation by revealing foot placement errors on the intact side.

The rung walking test has been shown to be sensitive to chronic movement deficits after adult and neonate lesions to the motor system, including rat models of stroke (Emerick and Kartje, 2004; Rieck-Burchardt et al., 2004; Farr et al., 2006; Ploughman et al., 2007), Parkinson’s disease (Metz and Whishaw, 2002; Faraji and Metz, 2007), and spinal cord injury (Z’Graggen et al., 1998; Merkler et al., 2000).  Furthermore, the ladder rung walking task detects changes in fine motor performance induced by physiological variables such as mild stress (Metz) and even changes in diet (Smith and Metz, 2005). In addition to rat models, the task is also useful for studying skilled walking in mice (Farr et al., 2006). Caution using this test needs to be used if severe spinal cord lesions are tested (Metz et al., 2000), as the rung walking task requires that animals are able to perform weight-bearing steps.

There is substantial evidence that animals with motor system lesion are able to compensate for lesion-induced deficits in skilled movements (Whishaw et al., 1997a,b, 1998; Miklyaeva et al., 1994; Kleim et al., 1998; Chu and Jones, 2000). Nevertheless, severe impairments may still be present. For example, animals with dopaminergic or red nucleus lesions recover the ability to walk on smooth surfaces or narrow beams, but measures of ground reaction forces reveal that their impairment is chronic (Muir and Whishaw, 1999, 2000). Because it reveals even subtle remaining motor deficits and compensatory adjustments, the rung walking test is a valuable tool for assessing loss and recovery of function due to brain or spinal cord injury, and the benefit of treatment approaches.
